# Uterine Endoplasmic Reticulum Stress and Its Unfolded Protein Response May Regulate Caspase 3 Activation in the Pregnant Mouse Uterus

**DOI:** 10.1371/journal.pone.0075152

**Published:** 2013-09-13

**Authors:** Arvind Suresh, Kalpana Subedi, Chandrashekara Kyathanahalli, Pancharatnam Jeyasuria, Jennifer C. Condon

**Affiliations:** 1 Department of Obstetrics and Gynecology, Magee Women’s Research Institute, University of Pittsburgh, Pittsburgh, Pennsylvania, United States of America; 2 Department of Cell Biology, Magee Women’s Research Institute, University of Pittsburgh, Pittsburgh, Pennsylvania, United States of America; Baylor college of Medicine, United States of America

## Abstract

We have previously proposed that uterine caspase-3 may modulate uterine contractility in a gestationally regulated fashion. The objective of this study was to determine the mechanism by which uterine caspase-3 is activated and consequently controlled in the pregnant uterus across gestation. Utilizing the mouse uterus as our gestational model we examined the intrinsic and extrinsic apoptotic signaling pathways and the endoplasmic reticulum stress response as potential activators of uterine caspase-3 at the transcriptional and translational level. Our study revealed robust activation of the uterine myocyte endoplasmic reticulum stress response and its adaptive unfolded protein response during pregnancy coinciding respectively with increased uterine caspase-3 activity and its withdrawal to term. In contrast the intrinsic and extrinsic apoptotic signaling pathways remained inactive across gestation. We speculate that physiological stimuli experienced by the pregnant uterus likely potentiates the uterine myocyte endoplasmic reticulum stress response resulting in elevated caspase-3 activation, which is isolated to the pregnant mouse myometrium. However as term approaches, activation of an elevated adaptive unfolded protein response acts to limit the endoplasmic reticulum stress response inhibiting caspase-3 resulting in its decline towards term. We speculate that these events have the capacity to regulate gestational length in a caspase-3 dependent manner.

## Introduction

Premature labor is the leading cause of neonatal mortality, however to date, few effective and broadly applicable interventions are available for the prevention of preterm birth [1]. A major hurdle in the development of treatment strategies is a poor understanding of the signaling mechanisms in the pregnant uterus that maintain myocyte quiescence during normal gestation [1]. We have proposed caspase-3 (CASP3) as a potential regulator of uterine quiescence [2]. Utilizing the mouse as our model system, we have also identified increased levels of active CASP3 at mid gestation, which decline as term approaches in a progesterone (P4) regulated manner through direct targeting of the uterine contractile architecture [2]. We also observed a gestationally regulated anti-apoptotic signaling cascade that permits the pregnant uterus to maintain a non-apoptotic phenotype despite elevated uterine CASP3 activity [3], [4].

Due to the potential importance of uterine CASP3 activity in regulating gestational length, this study has focused on determining the factors that may regulate uterine CASP3 during pregnancy with the hope that we can ultimately understand the mechanism by which uterine quiescence across is maintained across gestation, allowing pregnancies to be carried to tern. We speculate that by determining the factors that allow the pregnant uterus to transition from a quiescent to a contractile phenotype in normal term labor, that we may gain insight into the mechanisms that are perturbed allowing for the onset of pre-term birth. In this study we have defined that the uterine myocyte endoplasmic reticulum stress response (ERSR) and its related unfolded protein response (UPR) are the likely regulators of uterine myocyte CASP3 activity during pregnancy. Physiological stimuli such as fluxes in protein synthesis, cellular differentiation, hypoxia and glucose deprivation have been described to result in the accumulation of misfolded proteins and perturbation of ER homeostasis resulting in the activation of the ERSR and its adaptive UPR [5]–[7]. Physiological and pathophysiological processes such as aging, differentiation and a number of diseases including cancer, obesity, diabetes, neurodegeneration and viral infections have implicated the ERSR and the UPR as key regulators of cell death and/or survival through the modulation of caspase action [8]–[10]. During pregnancy, pathophysiological activation of the ERSR has been reported to play a role in placental dysfunction leading to intra uterine growth restriction and early-onset preeclampsia [11]–[15]. It has also been suggested that ER stress induced by oxidative stress in decidual cells may play a role in early pregnancy loss [16]–[18].

We propose that physiological and potential uterotonic stimuli that the pregnant uterus experiences across gestation may potentiate CASP3 activation through the ERSR thereby maintaining the pregnant uterus in a quiescent state. However, towards the end of pregnancy we suggest that increased activation of the adaptive UPR limits the uterine ERSR, thereby reducing CASP3 apoptotic potential resulting in its gestational decline towards term. Ultimately these events initially limit the uterine myocyte contractile potential from early to mid-gestation through CASP3 activation, however towards term they permit reconstitution of the uterine myocyte contractile architecture.

In summary this study examined three potential signaling pathways in the pregnant mouse uterus that may have the capacity to regulate CASP3 activity. Utilizing a gestational series of mouse uteri from E6–E19 we examined the presence of the ERSR in the pregnant uterus at the transcriptional and translational level. The traditional canonical intrinsic and extrinsic pathways, which initiate through transmembrane receptors at the cell surface or through the cytoplasmic release of the mitochondrial protein cytochrome C (CYCS), respectively [19], [20] were also examined as potential activators of uterine CASP3. While we found no evidence of activation of the extrinsic or intrinsic apoptotic-signaling pathways, robust gestationally mediated activation of the ERSR and UPR was observed in the pregnant mouse uterus across gestation. We therefore speculate that uterine CASP3 during pregnancy is activated and regulated in a non-apoptotic manner through the actions of the uterine ERSR and UPR.

## Materials and Methods

### Reagents

All chemicals were obtained from Sigma Aldrich, St Louis MO unless otherwise indicated. Recombinant mouse Bid (truncated and full length) (Cat# Pro-644) was purchased from ProSpec-Tany Technogene Ltd. (East Brunswick, NJ) and recombinant active caspase 8 (CASP8) (Cat# CC123) was obtained from Millipore Inc, (Billerca, MA).

### Animal Studies

All animal studies were approved by the Institutional Animal Care and Use Committee of the University of Pittsburgh. Timed-pregnant female CD-1 mice (6–8 weeks old) were obtained from Charles River and housed according to IACUC guidelines. Uterine tissues (n = 3 for each gestational time point) were harvested between 0800–1000 h on E6, E8, E10, E11, E12, E13, E15, E16, E17, E18 and E19. The uterine horn was cleared of all embryonic material and maternal decidua. The isolation of mitochondrial and cytosolic fractions, were performed on fresh uterine tissues, processed immediately after harvest as described below. For immunofluorescence studies, the uteri were fixed in 4% PFA overnight and subsequently embedded in paraffin blocks for sectioning. The remaining uterine tissue was washed in 1×PBS and flash frozen for subsequent protein and mRNA analysis.

### Protein Extraction

#### Extraction of cytoplasmic and nuclear protein fractions from frozen uterine tissues

Cytoplasmic and nuclear protein extracts were prepared from frozen uterine tissue as described previously [2]. Briefly, myometrial tissue (n = 3 for each gestational time point) was pulverized in liquid nitrogen, and homogenized in ice-cold NE1 buffer (10 mM Hepes (pH 7.5), 10 mM MgCl_2_, 5 mM KCL, and 0.1% Triton ×-100) and 1×protease/phosphatase inhibitor cocktail (Complete Mini EDTA free/PhosStoP) (Roche, Mannheim, Germany) using an IKA homogenizer (IKA Works Inc Wilmington, NC). The homogenate was centrifuged at 5000 RPM for 10 min at 4°C, and the supernatant was retained as the cytoplasmic fraction. The pellet was washed in NE1 and then resuspended in ice-cold NE2 buffer (25% glycerol, 20 mM HEPES (pH 7.9), 500 mM NaCl, 1.5 mM MgCl_2_, and 0.2 mM EDTA (pH 8.0)) with 1×protease/phosphatase inhibitor cocktail and was incubated on ice for 30 min, vortexed every 5 min, and centrifuged at 10,000 RPM for 10 min at 4°C. The supernatant was retained as the nuclear fraction. Our previous published data in the human myometrial cell [21] and in this current study of the pregnant mouse uterus, has revealed that the uterine cytoplasmic fraction (containing cytosol, membranes and subcellular organelles (excluding the nucleus)) displays stable levels of protein disulfide isomerase (PDI) across gestation and the nuclear levels of the nuclear receptor co-activator 3 (NCOA3) remain relative constant across gestation in the pregnant mouse uterus relative to subcellular protein concentrations. We have therefore utilized NCOA3 and PDI to ensure equal loading for immunoblotting and as markers to determine the purity of the isolated nuclear and cytoplasmic protein fractions, respectively. Subcellular protein concentrations were determined in triplicate by bicinchoninic acid protein (BCA) assay using the Pierce BCA Assay Kit (ThermoScientific Rockford IL).

#### Isolation of mitochondrial and cytosolic fractions from fresh uterine tissues

In order to isolate pure preparations of intact mitochondria and overcome problems associated with mitochondrial rupture in frozen tissues, we used freshly harvested uterine tissue for subcellular fractionation. The fresh mitochondrial and cytosolic fractions (intracellular fluid excluding all subcellular organelles) were isolated from uterine tissues using a protocol modified from previously described methods [22]–[24]. Freshly excised uterine tissue was homogenized in ice-cold isolation medium (320 mM Sucrose, 1 mM EDTA and 20 mM Tris-HCl buffer pH 7.1) in a Dounce-type glass homogenizer. The homogenate was centrifuged at 3500 RPM for 3 min at 4°C. The supernatant containing the crude cytosolic fraction was isolated and centrifuged at 10,000 RPM for 10 minutes at 4°C. The pellet containing the mitochondrial fraction was solubilized in radioimmune precipitation (RIPA) buffer containing 20 mM Tris-HCl (pH 7.5), 150 mM NaCl, 1 mM EDTA, 1% NP-40, 1% Triton-X 100. The supernatant was further centrifuged at 40,000 RPM to obtain the supernatant containing the pure cytosolic fraction. Known markers of the cytosolic fraction glyceraldehyde 3-phosphate dehydrogenase (GAPDH) and mitochondrial fraction, cytochrome c oxidase subunit 4 isoform 1 (COX4I1) were utilized to determine purity of the isolated fractions and as loading controls for immunoblot analysis. PDI is not a suitable loading control for cytosolic preparations as PDI is an ER associated protein. Subcellular protein concentrations were determined using the Pierce BCA Assay Kit (Thermo Scientific Rockford IL).

### Immunoblotting and Densitometric Analysis

Equal amounts of protein were loaded on NuPAGE pre-cast gradient gels (Life Technologies, Carlsbad CA) and transferred on to Hybond-P PVDF membranes (Millipore, Billerica MA). The membranes were blocked in 5% non-fat milk prepared in 1× Tris Buffered Saline with Tween-20 for 1 hr at room temperature and then incubated with the primary antibodies overnight. This was followed by incubation with horseradish peroxidase-conjugated secondary antibodies diluted in 5% milk-1×TBS-T buffer. Immunoreactive bands were visualized using an ECL detection system (ThermoScientific Rockford IL). The concentrations of primary antibodies and their sources are as follows, Cell Signaling Technologies (Beverly, MA)-HSPA5 (#3177 1:1000), DDIT3 (#5554 1: 200), full length and cleaved CASP12 (#2202 1:500), PDI (#3501 1:1000), full length and cleaved CASP3 (#9665 1:1000), COX4I1 (#4850 1:1000), GAPDH (#2118 1:2000), full length and truncated BID (#2003 1:500); Santa Cruz Biotechnology – CYCS (sc-7159 1:1000), XBP1 (sc-7160 1:500). Thermo Scientific – NCOA3 (#PA1-845 1:2000). The immunoreactive cytoplasmic, nuclear, cytosolic and mitochondrial bands obtained by immunoblotting were quantified using ImageJ (NIH Bethesda MD) and normalized to PDI, NCOA3, COX4I1 and CYCS, respectively as their protein concentrations were found to remain relatively unchanged in the pregnant mouse uterus across gestation.

### Immunofluorescence

Paraffin embedded uterine tissues were sectioned at 5 µm thickness and collected on Superfrost Plus slides (Fisher Scientific, Pittsburgh PA). Paraffin sections were deparaffinized in xylene and rehydrated through an alcohol series. For DDIT3 and HSPA5, antigen retrieval was performed by boiling the sections for 15 minutes in 10 mM citrate buffer pH 6.0. For cleaved CASP3 immunohistochemical analysis, uterine tissue sections were incubated with proteinase k (10 µg/ml) for 10 minutes at room temperature. Tissue sections were then blocked in 1 X PBS containing 5% normal donkey serum and incubated in the primary antibody overnight at 4°C at the following concentrations DDIT3 1:100, cleaved CASP3 (Cell Signaling #9664 1:100), HSPA5 1:100. Sections were then washed in 1×PBS and incubated in the fluorescent secondary antibody for 1 hr at room temperature. Donkey anti-rabbit secondary antibodies conjugated to either Alexa flour 488 or Cy3 (Jackson Immunoresearch, West Grove PA) were used at a dilution of 1∶500 in 1×PBS. The sections were then washed and incubated with 4′,6-diamidino-2-phenylindole (DAPI) to stain nuclei and mounted in gelvatol. Images were collected at 63× on a Leica DMRBE (Leica Microsystems Buffalo Grove IL) using a Q-Imaging Micro Publisher 5.0 RTV (QImaging Surrey, British Columbia Canada). Serial sections were used for immunoflourescence as differences in antigen retrieval methods for the CASP3 and DDIT3 antibodies precluded dual immunofluorescence analysis. Companion-blocking peptides were utilized to pre-absorb each primary antibody, at each gestational stage utilized in this study, thereby acting as negative controls for auto-fluorescence and false positives.

### RNA Isolation, cDNA Synthesis and Quantitative Real Time PCR

RNA was isolated from pulverized uterine tissues using the RNeasy Mini Kit (Qiagen Inc, Valencia CA) according to the manufacturer’s instructions. Total RNA was converted to cDNA using the High Capacity cDNA Reverse Transcription Kit (Life Technologies, Carlsbad CA). Primers were designed using NCBI Primer Blast to obtain amplicons from 50–170 bp. (See Table 1 for detailed primer information). Real Time Quantatative PCR (Q-PCR) was performed on the ABI 7900HT Sequence Detection System (Life Technologies Carlsbad CA) using a SYBR Green PCR Master Mix (Life Technologies Carlsbad CA). For each reaction, 40 ng of cDNA and a final primer concentration of 150 nM was used. *Rplp0* was used as a housekeeping gene as we have found *Rplp0* levels to remain stable across gestation in the pregnant mouse uterus [2]. Samples were assayed in triplicate (n = 3 for each gestational time point). Amplified mRNAs identified by Q-PCR were normalized to *Rplp0*. Expression data were analyzed using the ΔΔCt method [25].

**Table 1:** List of Primers Used for QPCR.

Gene	GenBankAccession#	Fwd Primerstart	Rev PrimerStart	Fwd PrimerSequence	Rev PrimerSequence
*Ddit3*	NM_007837.3	635	758	ggctctgatcgaccgcatgg	gcagtgcagggtcacatgctt
*Casp12*	NM_009808.4	891	1022	gtcggagtctgagaaacaaacccaa	agctcaacacacgttcctcatctg
*Hspa5*	NM_001163434.1	272	426	ctgctgaggcgtatttgggaaaga	tgcttgtcgctgggcatcattg
*Rplp0*	NM_007475.5	525	624	acctccttcttccaggcttt	cccaccttgtctccagtcttt
*Atf6*	NM_001081304.1	1552	1713	ccgcattctccagggtgctct	ctcctgcggatggcgtcaaag
*Atf4*	NM_009716.2	735	824	tgggttctccagcgacaaggc	gcatcctccttgccggtgtct

A list of the primers used for mRNA expression analysis of genes in the ER Stress and Unfolded Protein Response in the pregnant mouse uterus across gestation.

### Statistical Analysis

All data are representative of at least three independent experiments. Immunoblots were analyzed by densitometry using ImageJ (NIH, Bethesda MD). Statistical analysis of immunoblots and Q-PCR were performed with StatPlus:mac software 2009 version (AnalystSoft Inc.). Data are represented as mean ± standard error (SE) of the mean. The results were subjected to a one-way ANOVA followed by pairwise comparison (Student-Neuman-Keuls method) to determine differences between groups. Significance was set at P<0.05.

## Results

### Gestational Regulation of CASP3 Expression in the Pregnant Mouse Uterus

**Figure 1:**
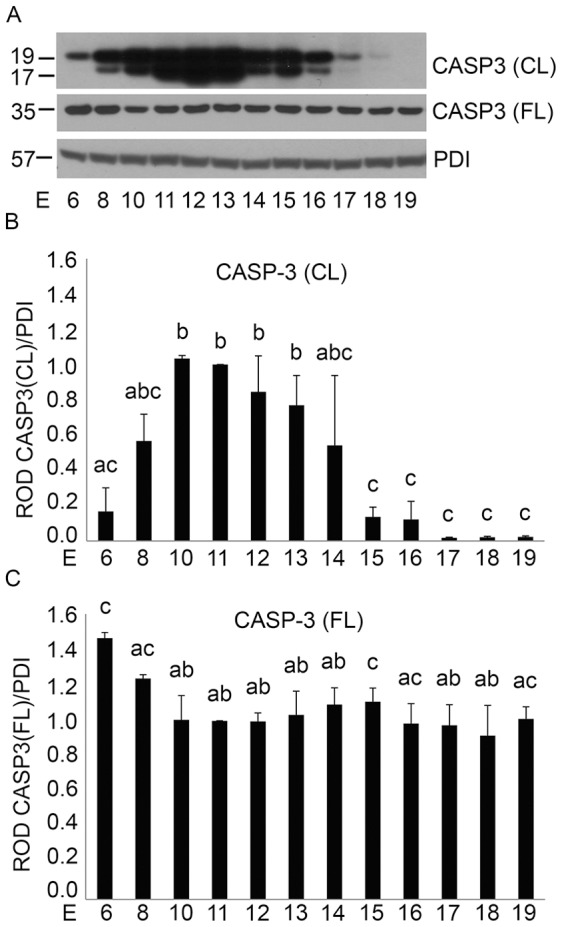
Gestational profile of uterine CASP3 activation in the pregnant mouse uterus from E6–E19. A) Representative western blot of a gestational series of mouse uteri immunoblotted for cleaved (CL) and full length (FL) CASP3 protein (n = 3 for each time point). Relative optical density (ROD) of B) cleaved and C) full length CASP3 normalized to the loading control, PDI. Data are represented as Mean ROD ± SE. N = 3 for each time point. Data labeled with different letters are significantly different from each other (P<0.05).

Cytoplasmic proteins were isolated from uterine tissues of pregnant mice from gestation E6 to E19 (n = 3 for each gestational time point) as described in the Materials and Methods. To determine the levels of full length and active CASP3 immunoblotting was performed (Fig. 1). As seen in the immunoblots of Figure 1A levels of cleaved CASP3 seen as a doublet at 19 and 17 kDa were minimal early in gestation and exhibited an increase through E10–E13, which gradually declined to barely detectable levels towards the end of gestation. In contrast levels of full length CASP3 remain constant across gestation. Both full length and cleaved CASP3 were normalized to the 57 kDa protein PDI (Fig. 1A), which remained stable throughout gestation. In the densitometric analysis outlined in Figure 1B, cleaved CASP3 exhibited a 5–6 fold increase through E10–E13 while full length CASP3 levels (Fig. 1C) were significantly higher earlier in gestation at E6 and E8 with an approximate 0.5 fold decline in full length CASP3 levels from E8 to E10 which was maintained at most gestational time points to term. Our previously published mouse uterine CASP3 immunoblots, failed to include the early gestational time points of E6, 8 and 10 [2]. The inclusion of these earlier gestational timepoints revealed the timing of the initial activation of CASP3 cleavage resulting in a significant decrease in full length CASP3.

### Uterine CASP3 Activation does not Occur Through the Extrinsic Caspase Activation Pathway

**Figure 2:**
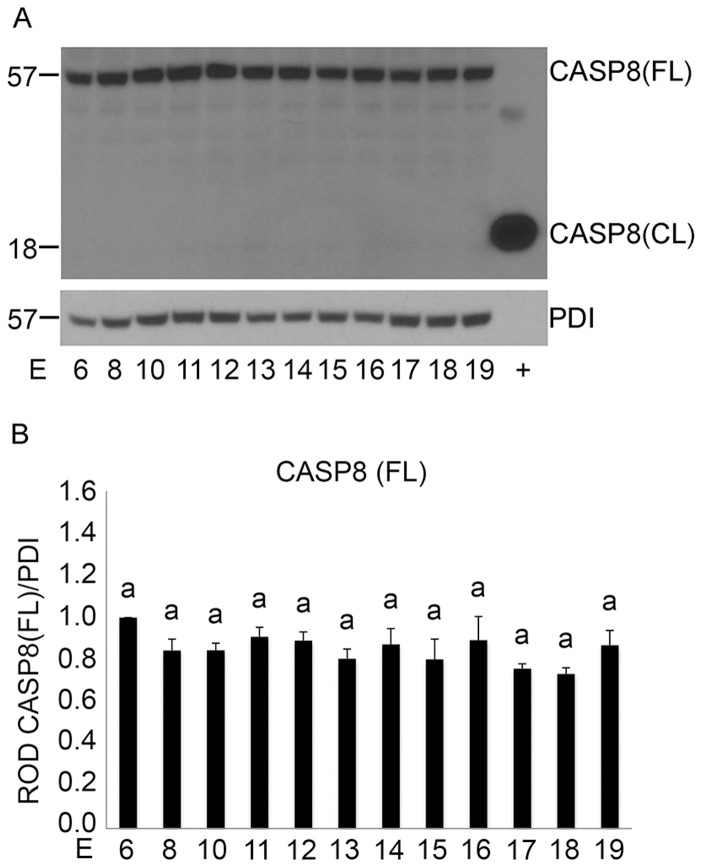
Examination of uterine CASP8 cleavage across gestation from E6–E19. A) Western blot analysis of the cytoplasmic fraction in a gestational series of mouse uteri immunoblotted for full length (FL) and cleaved (CL) CASP8. Recombinant active CASP8 was utilized as a positive control (+) and PDI was used as a loading control B) Graphs of ROD of full length CASP8 normalized to PDI. Data are represented as Mean ROD ± SE. N = 3 for each time point.

The hallmark of an active extrinsic caspase signaling pathway is the appearance of cleaved CASP8 and tBID [19], [26]. Therefore in order to determine if activation of uterine CASP3 during pregnancy occurs through the extrinsic caspase activation cascade we first examined the levels of both full length (57 kDa) and cleaved CASP8 (18 kDa) in the pregnant mouse uterus across gestation. As can be seen in Figure 2A, full length CASP8 remains intact across gestation while the cleaved active form of CASP8 at 18 kDa represented in our positive recombinant control remains absent. Densitometric analysis of full length cytoplasmic CASP8 normalized to PDI (Fig. 2B) failed to demonstrate a significant difference in levels across gestation (n = 3).

**Figure 3:**
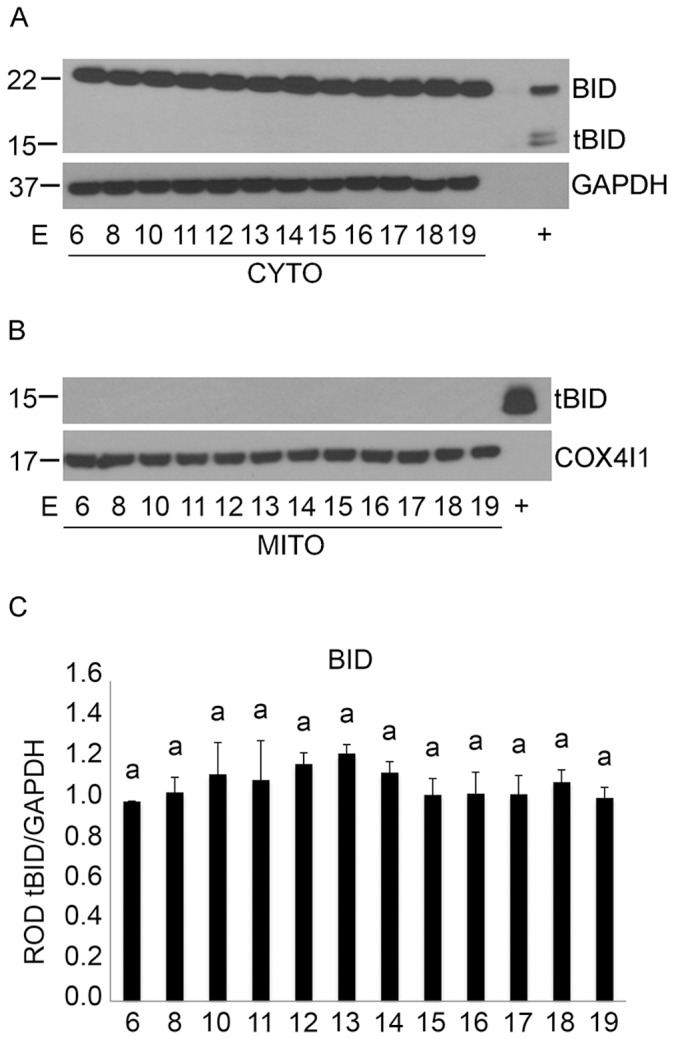
Examination of BID cleavage and translocation in the pregnant mouse uterus from E6–E19. Western blot analysis of BID and tBID in the cytosolic fraction (A) and tBID in the mitochondrial fraction (B). GAPDH and COX4I1 were utilized as a loading control for the cytosolic and mitochondrial fraction respectively. C) Graphs of ROD of BID normalized GAPDH. Data are represented as Mean ROD ± SE. N = 3 for each time point.

Downstream from CASP8 in the extrinsic pathway of caspase activation is the cleavage of the Bcl-2 family protein, BID [19], [20], [26]. Truncated BID (tBID) translocates to the mitochondrial fraction and initiates the intrinsic caspase activation pathway [26]. To examine whether tBID migrates from the cytosol to the mitochondria, protein fractions were isolated from freshly harvested uterine tissue by differential centrifugation and subjected to immunoblotting analysis. As can been seen in Figure 3A, though full length BID at 22 kDa was present in the cytosol at each gestational timepoint, tBID was only detectable in our recombinant positive controls at15 kDa. A faint band in Figure 3A between E19 and our positive control represents minor spillover from our recombinant BID positive control lane. Figure 3B clearly demonstrates that the mitochondrial fraction also remains void of tBID confirming the absence of extrinsic caspase pathway activation. The mitochondrial protein COX4I1 and the cytosolic protein GAPDH were utilized as our loading and fraction purity controls. Densitometric analysis of full length cytosolic BID (Fig. 3C) normalized to GAPDH showed no significant difference in the levels of intact full length BID across gestation (n = 3 for each gestational time point).

### Uterine CASP3 Activation does not Occur Through the Intrinsic Caspase Activation Pathway

**Figure 4:**
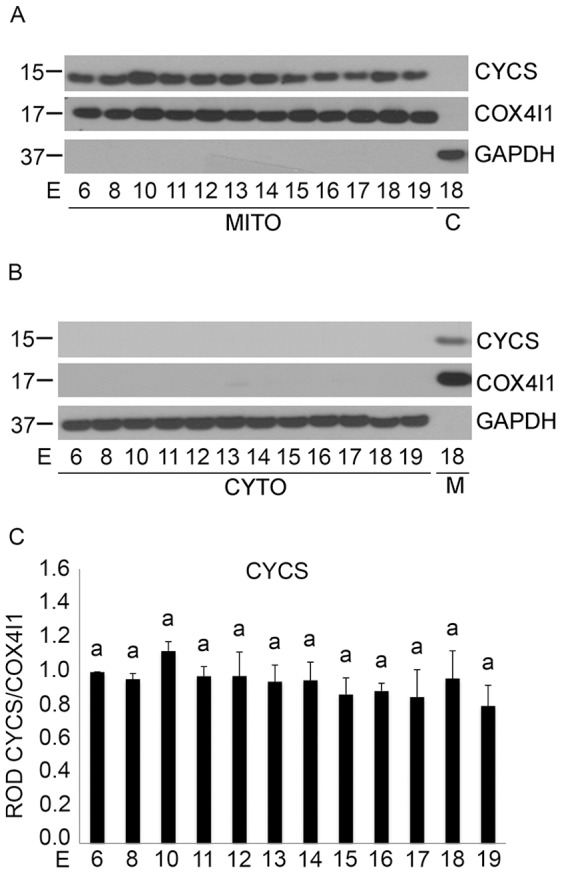
Mitochondrial CYCS retention in pregnant mouse uterus from E6–E19. Representative western blots of a gestational series of mouse uteri immunoblotted for A) CYCS in the mitochondrial fraction and B) in the cytosolic fraction. C) ROD of CYCS in the mitochondrial fraction normalized to COX4I1. Levels of GAPDH and COX4I1 were used as controls to determine the loading and purity of the cytosolic and mitochondrial fractions respectively. Data are represented as Mean ROD ± SE. N = 3 for each time point.

To examine whether uterine CASP3 activation occurred through the intrinsic caspase activation pathway, we determined whether the pregnant uterine myocytes released the 15 kDa CYCS from the mitochondria to the cytosol indicating a loss of mitochondrial integrity [19] and resulting in potential CASP3 activation through apoptosome formation [19]. Immunoblotting analysis determined that levels of mitochondrial CYCS remained unchanged throughout pregnancy (Fig. 4A). The absence of cytosolic CYCS from E6–E19 indicated the successful retention of CYCS in the mitochondria across gestation. GAPDH and COX4I1 were also used to assess equal loading and purity of the mitochondrial and cytosolic fractions respectively. Densitometric analysis revealed no significant changes in mitochondrial CYCS levels across gestation in the pregnant mouse uterus (Fig. 4C) (n = 3 for each gestational timepoint). Taken together the data represented in Figures 2, 3 and 4 suggested that neither the intrinsic nor the extrinsic caspase activation cascade was involved in uterine CASP3 activation (Fig. 1).

### Endoplasmic Reticulum Stress Induced Caspase Activation is Gestationally Regulated in the Pregnant Mouse Uterus

**Figure 5:**
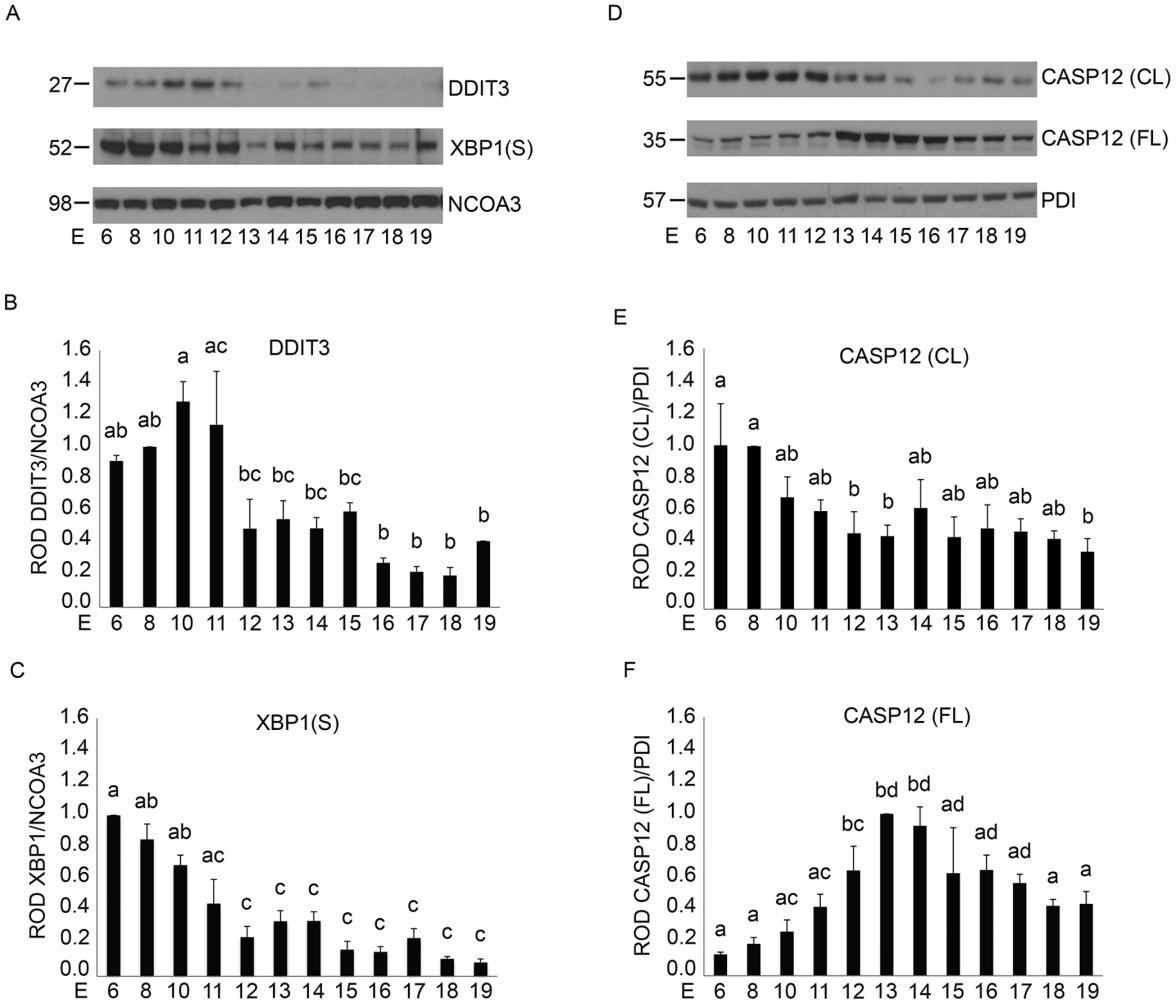
Gestational regulation of ER stress signaling in the pregnant mouse uterus from E6–E19. A) Analysis of protein expression of DDIT3 and XBP1(S) in the nuclear fraction of a gestational series of mouse uteri from E6–19. NCOA3 was used as a loading control. Graphs of ROD of B) DDIT3 and C) XBP1(S) normalized to NCOA3. D) Representative western blots of cleaved (CL) and full length (FL) CASP12 in the cytoplasmic fraction across gestation. PDI was used as a loading control. ROD of E) CL CASP12 and F) FL CASP12 normalized to PDI. Data are represented as Mean ROD ± SE. N = 3 for each time point. Data labeled with different letters are significantly different from each other (P<0.05).

Cytoplasmic and nuclear protein extracts isolated from pregnant mouse uterine tissues from E6–E19 were examined for markers of the ERSR by immunoblotting. The presence and gestational regulation of the transcription factor DDIT3, a marker for activation of ERSR [10] was identified in the pregnant uterus (Fig. 5A). DDIT3 levels in the nuclear fraction were maximal from E6–E11 and declined towards term. DDIT3 levels at E6 to E11 represented a two-fold increase over E12–E15 and approximately a four-fold increase over E16–E19 (Fig. 5B). The spliced isoform of XBP1 (XBP1(S)) found at 52 kDa in the nuclear fraction, is a proximal marker of ER stress [27]. XBP1(S) protein levels analyzed by immunoblot analysis revealed a marked elevation early in gestation (Fig. 5A) which declined towards term. Densitometric analysis indicated that XBP1(S) levels from E6–E11 were 2.7 fold higher than E12–E15 and approximately 5 fold higher than levels from E16–E19 (Fig. 5C, n = 3 for each gestational timepoint). Subsequently, we examined the activation profile of the initiator caspase, CASP12, an ER resident caspase in the pregnant mouse uterus [10] which has previously been associated with CASP3 activation through the ERSR [28]. Analysis of active CASP12 revealed in a similar manner to both DDIT3 and XBP1(S), the appearance of elevated levels of the cleaved active form of uterine CASP12 early in gestation from E6–E11 (Fig. 5D) with a reciprocal pattern observed for full length CASP12. Densitometric analysis of cleaved and full length cytoplasmic CASP12 normalized to PDI (Fig. 5E and 5F) indicated an approximate two-fold increase in the cleaved form at E6–E11 over E12–E19 (Fig. 5E). In contrast, full length CASP12 increased towards term where between E12–E15 full-length CASP12 levels were three-fold higher than levels at E6–E11 and 1.5-fold higher than E16–E19 (Fig. 5F). NCOA3 was utilized as a nuclear protein loading control as it was found to remain relatively stable through gestation in the pregnant mouse uterus [29].

### Activation of the Adaptive Unfolded Protein Response in the Pregnant Mouse Uterus

**Figure 6:**
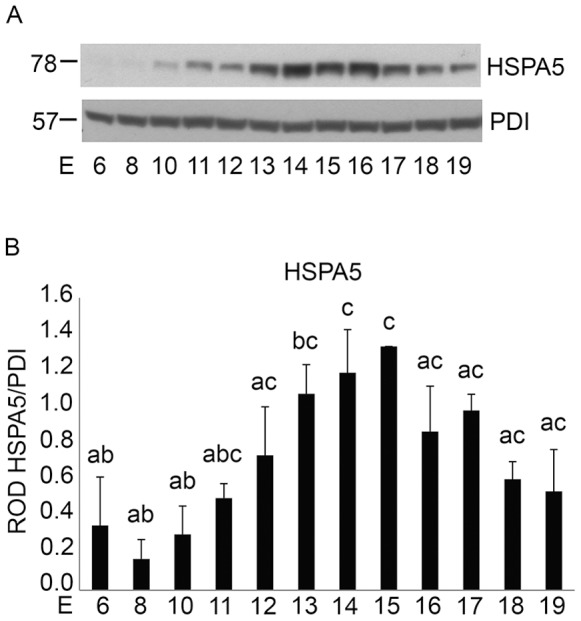
Gestational regulation of the UPR marker HSPA5 in the pregnant mouse uterus from E6–E19. A) Analysis of protein expression of HSPA5 in the cytoplasmic fraction of a gestational series of mouse uteri. PDI was used as a loading control. B) Graph of ROD of HSPA5 normalized to PDI. Data are represented as Mean ROD ± SE. N = 3 for each time point. Data labeled with different letters are significantly different from each other (P<0.05).

The expression of the chaperone HSPA5, a marker of the adaptive UPR [30] was analyzed in the cytoplasmic fractions of uterine tissues across gestation by western blotting (n = 3 for each gestational timepoint). As observed in Fig. 6A activation of the UPR as indicated by increased levels of the chaperone protein HSPA5 occurs in gestationally regulated manner increasing gradually from E8 to E15 and subsequently declining towards term. Utilizing PDI as our loading control densitometric analysis demonstrated a 3.2-fold induction of HSPA5 E12–E15 over E6–E11 levels (Fig. 6B).

### Localization of DDIT3, CASP3, HSPA5 and PDI in the Pregnant Mouse Myometrium

**Figure 7:**
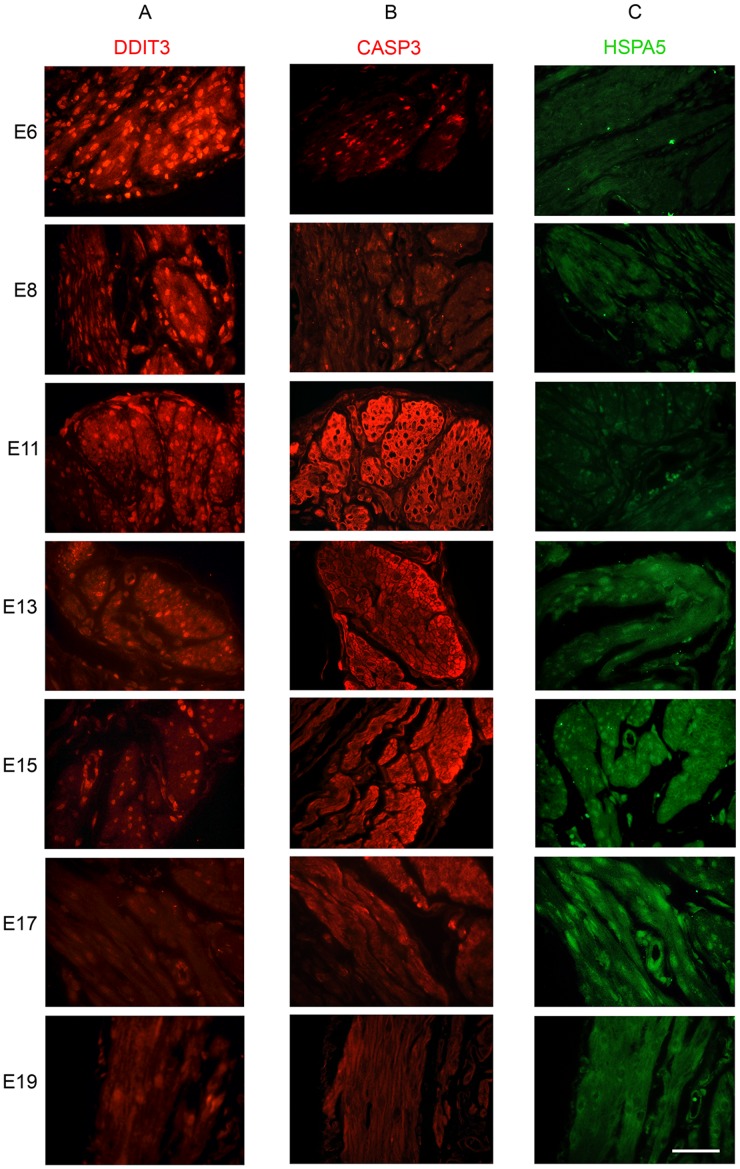
Localization of ERS and UPR markers in the pregnant mouse uterus. Immunoflouresence studies were used to determine the localization of A) active CASP3 (Red) B) DDIT3 (Red) C) and HSPA5 (Green) at E 6, 8, 11, 13, 15, 17 and 19.

To determine the localization of DDIT3, active CASP3 and HSPA5 in the pregnant mouse uterus across gestation (n = 3 for each gestational timepoint examined), we performed immunofluorescence analysis on formaldehyde fixed paraffin embedded sections of mouse uterine tissues as described in the Materials and Methods. Immunohistochemical analysis of DDIT3 confirmed its nuclear localization in uterine myocytes of the pregnant mouse uterus (Fig. 7A) and though these analyses are not quantitative it is clear that early in gestation, uterine myocyte DDIT3 levels are more abundant than at later gestational timepoints. We confirmed that cleaved uterine CASP3 was also confined to the myometrial compartment of the pregnant uterus and paralleled DDIT3 levels in that intense immunofluorescence was observed for cleaved CASP3, early in gestation and declining towards term (Fig. 7B). Immunoreactive HSPA5 (Fig. 7C) was observed in the ER of the uterine myocytes.

### Transcriptional Regulation of the ER Stress and Unfolded Protein Response in the Pregnant Mouse Uterus during Pregnancy

**Figure 8:**
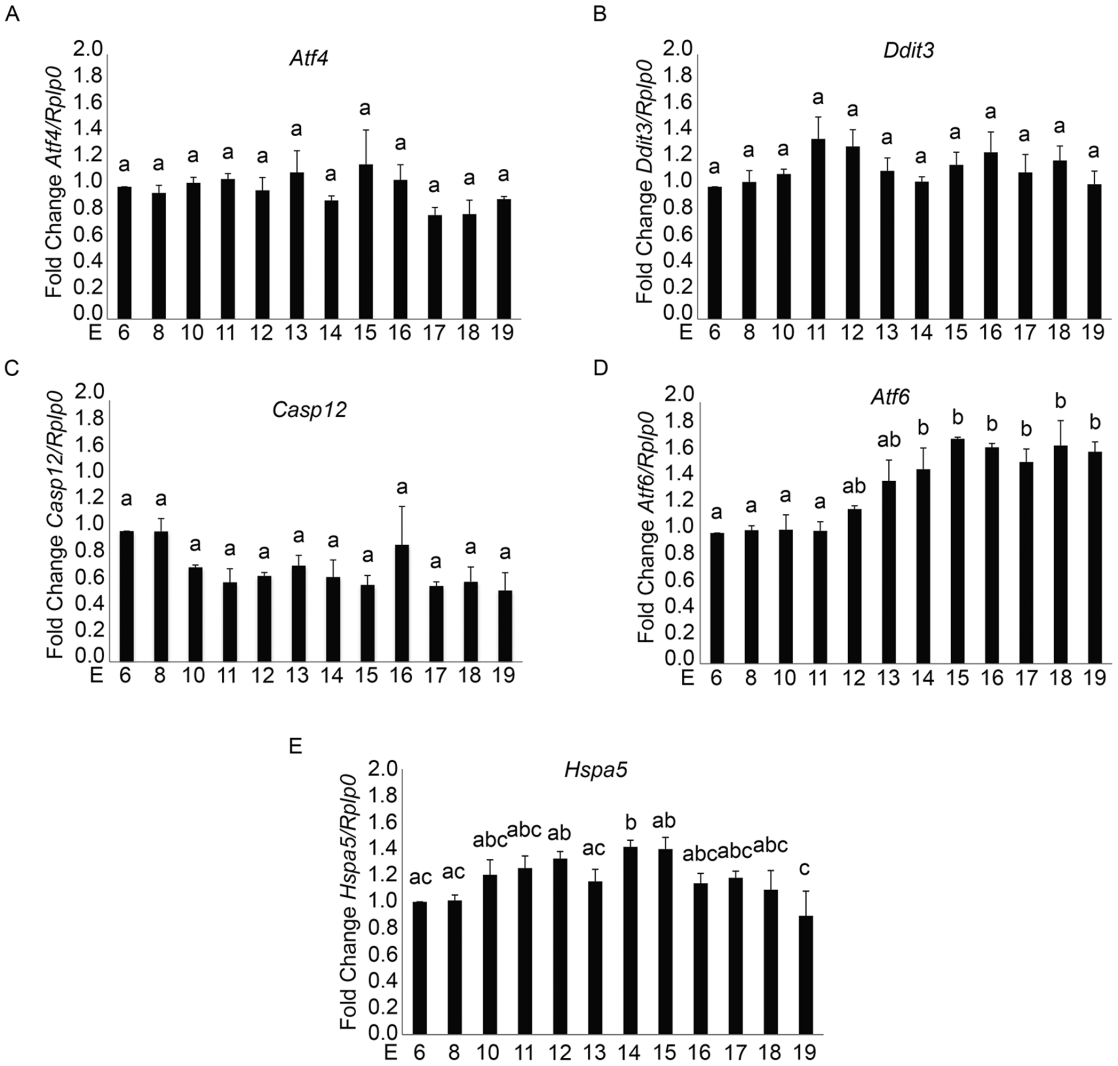
Transcriptional regulation of the ERSR and UPR in the pregnant mouse uterus from E6–E19. Gestational regulation of A) *Atf4,* B) *Ddit3*, C) *Casp12,* D) *Atf6* and E) *Hspa5* in the pregnant mouse uterus from E6–19 as analyzed by Q-PCR. Expression was normalized to *Rplp0,* which has been found to remain constant across gestation. Data are represented as mean fold change in expression ± SE. N = 3 for each time point. Data labeled with different letters are significantly different from each other (P<0.05).

Examination of the mRNA levels of *Atf4*, *Ddit3, Casp12, Atf6* and *Hspa5* across gestation in the pregnant mouse uterus was performed utilizing Q-PCR analysis. ATF4 is a transcription factor that is involved in the induction of *Ddit3* expression occurring as a result of ER stress [6], [7], [10]. As can be observed in Fig. 8A and 8B, the expression of *Atf4* and *Ddit3* transcript did not significantly change through the duration of pregnancy. Levels of full length *Casp12* demonstrated approximately a 1.4-fold induction at E6 and E8 in comparison with the rest of gestation but this increase was not statistically significant (Fig. 8C). We also analyzed transcript levels of *Atf6,* which has been shown to be involved in the induction of *Hspa5* expression [6], [7], [10]. We found that *Atf6* transcript showed approximately a 70% induction at E13–19 in comparison with E6–12, which was statistically significant (Fig. 8D). *Hspa5* expression showed a statistically significant 1.5 fold induction at E15 in comparison with earlier and later gestational time points (8E). Due to poor sensitivity of commercially available antibodies we were unable to perform immunohistochemical or immunoblotting analysis for ATF4 or ATF6.

## Discussion

We hypothesize that activation of the ERSR and the resulting UPR in the uterus are central in the non-apoptotic triggering and management of uterine CASP3 activity during pregnancy. We have previously determined that uterine CASP3 activation during pregnancy may have the capacity to effectively regulate the timing of parturition, through modulation of the uterine myocyte contractile architecture [2]. The objective of this study was to examine the mechanism by which uterine CASP3 is maintained in order to prolong uterine quiescence across gestation and on the other hand define the mechanism, which disables CASP3′s apoptotic potential and removes CASP3 from the uterine myocyte in a gestationally regulated manner to permit a successful contractile response at term.

The first indication that ER stress may act as a potential mechanism controlling uterine CASP3 came from the observation that although we had robust CASP3 activity we were unable to detect its mechanism of activation through either the classic intrinsic or extrinsic apoptotic signaling pathways (Fig. 2, 3 and 4). The extrinsic pathway of CASP3 activation is initiated by death ligand binding to the trans-membrane death receptor of the tumor necrosis factor receptor type 1 superfamily [31], which triggers CASP8 cleavage and activation. CASP8 once activated can directly cleave and mediate CASP3 activation or indirectly via the cleavage of the pro-apoptotic molecule BID and its translocation to the mitochondria resulting in CYCS release to the cytoplasm. As demonstrated in Figure 2 and 3 despite the robust CASP3 activation observed in Figure 1 there is no evidence for CASP8 cleavage or truncation of tBID in the pregnant mouse uterus across gestation. The intrinsic caspase activation pathway which is utilized to eliminate cells in an apoptotic response to stimuli such as ionizing radiation, chemotherapeutic drugs, mitochondrial damage and certain developmental cues [19], [32], [33] was also examined in the pregnant uterus. The intrinsic pathway leads to the release of CYCS from the mitochondria [34] resulting in formation of the apoptosome and CASP3 activation. As can be observed in Figure 3 and 4 there is no evidence of increased stimulation of the intrinsic apoptotic pathway in the pregnant mouse uterus. This is signified by the absence of tBID mitochondrial translocation, and the failure to release CYCS from the mitochondria to the cytosol at any gestational timepoint examined.

Recently an alternate, mitochondrial independent mechanism of CASP3 activation has been described, where CASP3 is cleaved and activated as a direct result of ER resident CASP12 activation. [28] The ER is an organelle that has essential roles in multiple cellular processes that are required for normal cell function and survival. These processes include intracellular calcium homeostasis, protein secretion and lipid biosynthesis [35]–[37]. One of the functions of the ER is to correctly fold and assemble proteins prior to their transit to intracellular organelles and the cell surface [38]. However, efficient protein folding requires a stable luminal environment [35]. Therefore the ER is exquisitely sensitive to alterations in homeostasis and any perturbation can lead to an accumulation of misfolded proteins and an induction of the ERSR. The ERSR signal can act as a double-edged sword for the cell and for normal physiology. Alternate adaptation strategies via the UPR can generate a spectrum of context-dependent cellular consequences ranging from cell recovery to apoptotic cell death [7], [30], [39], [40]. CASP12/(CASP4 in the human) activation occurs in response to the cell undergoing ER stress and hosting an UPR [41]–[47]. As is shown in Figure 5 the hallmarks of the ERSR were clearly detectable in the pregnant mouse uterus from E6 to E19. The appearance of the DDIT3 and XBP1(S) suggest that the pregnant uterus experiences a prolonged period of ER stress from early in gestation. The induction of prolonged pro-apoptotic DDIT3 activation via the ERSR has the capacity to promote apoptotic cell death via CASP12 and CASP3 activation. Accordingly in the pregnant uterus concomitant with the elevated levels of uterine DDIT3 we observe robust activation of CASP12 and CASP3 (Fig. 5D and Fig. 1A). However we discovered that the XBP1(S)/DDIT3 mediated increase in uterine CASP3 is transitory and declines towards the end of gestation. Taken together these data suggest that though uterine ERSR activates CASP3 via DDIT3 action, the apoptotic signal is actively resolved. Our transcriptional analysis of *Atf4, DditT3 and Casp12* in the pregnant mouse did not reveal any significant changes across gestation (Fig. 8), confirming the importance of the post transcriptional and post-translational events previously linked with the activation of these ERSR associated molecules in other tissues [30], [48]. We propose that resolution of the ERSR is likely mediated through the induction of the pro-survival, anti-apoptotic adaptive arm of the UPR (Fig. 6). Chaperone proteins such as HSPA5 which permit increased protein folding and export, permitting the ER to regain homeostasis [7], [30] are highly upregulated in the pregnant mouse uterus and reach maximal levels at E14 [49] (Fig. 6). We also observed that changes in *Hspa5* mRNA levels across gestation (Fig. 8) mirrored the changes in HSPA5 protein levels (Fig. 6). We also found an increase in the transcriptional levels of *Atf6*, which has been shown to induce *Hspa5* expression. Elevated levels of HSPA5 assist in clearing the backlog of accumulated proteins thereby in the context of the pregnant uterus suggest an active resolution of the uterine ERSR occurs towards term. Immunohistochemical analysis seen in Fig. 7 confirmed the localization and gestational profile of DDIT3, CASP3 and HSPA5 in the uterine myocytes of the pregnant mouse uterus.

**Figure 9:**
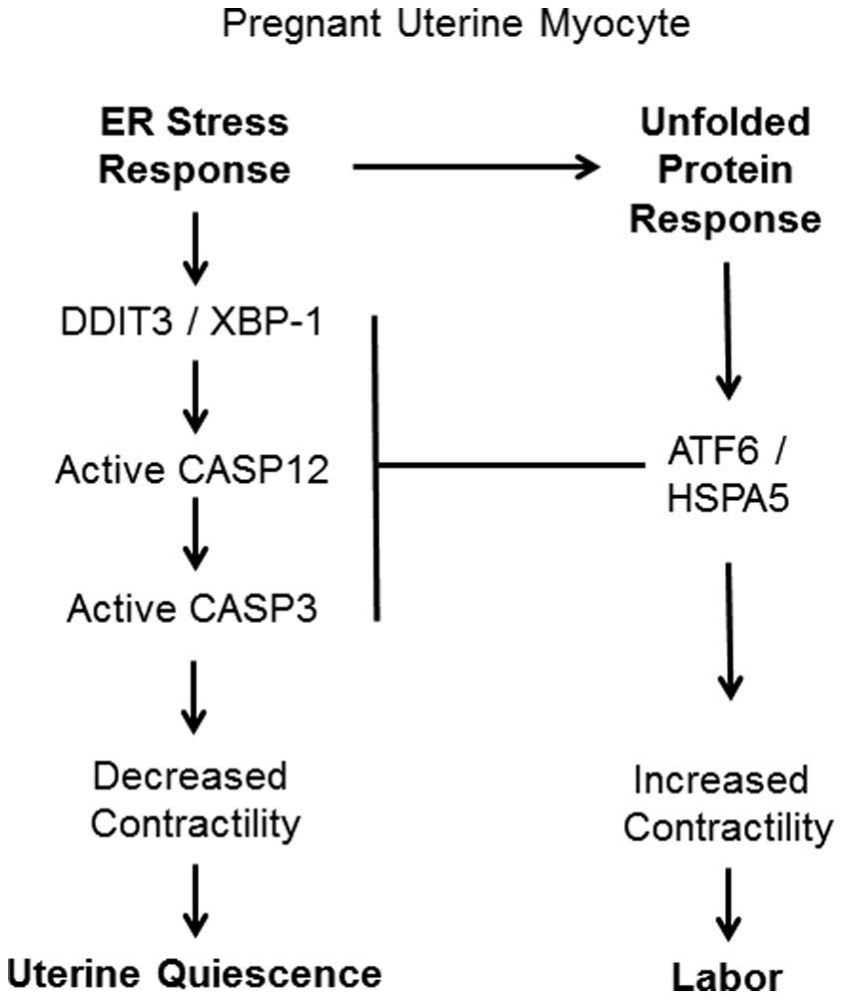
A model representing the potential role of ERSR and the UPR in the gestational regulation of uterine CASP3 activation and inhibition.

In summary, we propose that throughout pregnancy the uterine myocyte must monitor, tolerate and adapt to intrinsic and extrinsic uterotonic stimuli [50], [51]. We propose that the pregnant uterine myocyte utilizes physiological and uterotonic events as signals indicating the need to activate the potential tocolytic enzyme CASP3 in order to maintain myocyte quiescence. Activation of uterine CASP3 during pregnancy occurs in a non-apoptotic manner and this study proposes that the ER of the pregnant uterine myocytes hosts and manages this adaptive response through activation of an ERSR and triggering of the UPR. We suggest that prolonged pregnancy related myometrial ER stress as outlined in Figure 9, results in the induction of the pro-apoptotic protein, DDIT3 is associated with the activation of CASP12 (CASP4 in the human), which in turn cleaves and activates CASP3, independent of mitochondrial release of CYCS [28], [41]–[45]. However in the context of adaptation and survival, the chaperone proteins such as HSPA5 are elevated, which allows for increased protein folding and export [30], permitting the ER to regain homeostasis resulting in decreased ERSR and a withdrawal of uterine CASP3 towards term. Decreased CASP3 levels allow the pregnancy uterine myocyte regain is contractile phenotype towards term. Understanding how the uterine myocyte through the UPR can allow for adaptation, instead of apoptosis is of tremendous physiological importance, in the context of uterine quiescence and the pregnant uterus. Our future directions include defining the potential hormonal regulators of the uterine ERSR and UPR during pregnancy and defining the relevance of the ERSR and UPR to the maintenance of gestational length by manipulating these responses *in vivo*. We speculate that in the instances of pre-term birth an inappropriate ERSR or an inability to host an appropriate UPR may trigger a precocious withdrawal of uterine CASP3 allowing for a premature increase in uterine contractile responsiveness. With the growing recognition of an association of ER stress with human disease and with increased understanding of the fundamental mechanisms regulating ER stress, novel targets for drug discovery and new strategies for therapeutic preventative intervention are beginning to emerge and may ultimately be utilized to reveal indicators for and the resolution of pre-term birth.
